# Recurrence rate with inferior conjunctival autograft transplantation compared with superior conjunctival autograft transplantation in pterygium surgery: a meta-analysis

**DOI:** 10.1186/s12886-021-01889-4

**Published:** 2021-03-09

**Authors:** Wenwei Li, Yaoyong Lou, Bin Wang

**Affiliations:** Department of Ophthalmology, Tongde Hospital of Zhejiang Province, 234 Gucui Road, Hangzhou, 310012 China

**Keywords:** Pterygium, Inferior conjunctival autograft, Superior conjunctival autograft, Recurrence

## Abstract

**Background:**

Conjunctival autograft transplantation from superior conjunctiva is often chosen to lower the postoperative recurrence rate for pterygium treatment. However, inferior conjunctival autograft (ICA) might be taken as an alternative surgery method, especially under certain conditions. Consequently, we designed this research to estimate and contrast the result of inferior conjunctival autograft and superior conjunctival autograft (SCA) on the postoperative recurrence rate.

**Methods:**

We searched through network database (PubMed, Embase and Cochrane Central Register of Controlled Trials) to choose suitable randomized controlled trials (RCTs). Based on Cochrane review methods, we evaluated eligibility and risk of bias of included studies. The primary measures included postoperative recurrence rate. Pooled risk ratios (RRs) and 95% confidence intervals (CIs) were assessed. RevMan 5.3 software was utilized to conduct statistical analysis.

**Results:**

Four RCTs composed of a total of 438 eyes were included in this meta-analysis, with 234 eyes in the inferior conjunctival autograft group and 204 eyes in the superior conjunctival autograft group. Statistical meta-analysis revealed that the postoperative recurrence rate was similar between the two groups (RR = 0.77, 95% CI: 0.36 to 1.62, *P* = 0.49). Only two RCTs applied the postoperative pain scale and one of them did not provided adequate numbers.

**Conclusions:**

Our meta-analysis indicated that inferior autograft transplantation and superior autograft transplantation had a similar effect on postoperative recurrence rate. The inferior autograft group might have a less postoperative pain. Subsequent RCTs which have more patients participated and more outcomes are needed to confirm our conclusions in years to come.

**Supplementary Information:**

The online version contains supplementary material available at 10.1186/s12886-021-01889-4.

## Background

Pterygium is a kind of ocular surface disease frequently seen in ophthalmology clinic, which is defined as a fibrovascular growth extending from the conjunctiva onto the nasal, temporal, or both sides of the cornea. Generally it was thought to be a degenerative condition, characterised by abnormal subconjunctival fibrovascular growth onto the cornea. Nevertheless, in last several years, some researches have indicated that due to the dysfunction of local limbal stem cells, pterygium is a kind of ocular surface disorder, which has a close relationship with proliferation and inflammation. It often leads to chronic ocular irritation, compromised vision, and unsatisfactory appearance [1]. Although medical treatment with drugs such as cyclosporine often alleviates symptoms and obviates or delays surgery, surgical excision remains the main treatment for pterygium. However, recurrence remains the major challenge that has been troubling for patients and ophthalmologists. Recurrence rate after surgical removal of pterygium can be as high as 24 to 89% in some operations [2]. Among the numerous different surgery methods, conjunctival autograft transplantation after pterygium excision has been proved to be the optimal solution with a low recurrence rate and high safety by a lot of researchers. Gómez-Márquez first described a free conjunctival autograft to cover a bare scleral defect after pterygium excision in 1931 [3]. However, until 35 years ago, Kenyon proposed the current conjunctival autograft technique in the pterygium surgery. From then on, a lot of follow-up studies have been designed and confirmed that it is safe and effective in reducing the number of recurrences [4–8].

In general, we collect the conjunctival graft from the superior bulbar conjunctiva during the operation. However, when faced with patients who have conjunctival scarring of the superior conjunctiva or who are potentially diagnosed with glaucoma and may require future filtration surgery, the inferior bulbar conjunctiva could be taken as an alternative to be selected for surgery [9, 10]. Nevertheless, there is insufficient knowledge available about the recurrence of a graft harvested from the inferior bulbar conjunctiva. By far, only several studies have been conducted and compared the recurrence rates associated with the use of inferior conjunctival autograft (ICA) and superior conjunctival autograft (SCA) in pterygium surgery [9, 11–14]. As far as we know, there is no meta-analysis on comparison of the recurrence rates of these two surgery methods. Therefore, a meta-analysis is essential to assess the recurrence rate of inferior conjunctival autograft compared with superior conjunctival autograft for pterygium.

## Methods

### Search strategy

Comprehensive literature search was performed on 3 databases, including PubMed, Embase and Cochrane Central Register of Controlled Trials on comparison of the recurrence of inferior conjunctival autograft versus superior conjunctival autograft for pterygiums. The keywords used in the search were “pterygium”, “conjunctiva”, “autograft”, “inferior” and “superior”. Date or language restrictions were not applied during the search. Other search for additional studies was made by contacting other sources or reviewing references of included studies manually. The final search was performed on February 24, 2020 (See Additional file 1).

Two reviewers (WWL and BW) independently conducted the database searches and browsed the abstracts. And then, full-text articles were assessed for eligibility by reading to determine whether the articles met the inclusion and exclusion criteria. A third reviewer (YYL) was consulted when disagreement existed.

### Inclusion and exclusion criteria

The inclusion criteria were as follows: (1) RCTs involving primary pterygium patients surgery; (2) studies comparing inferior conjunctival autograft versus superior conjunctival autograft; (3) studies in which outcome measures included the recurrence rate. Exclusion criteria were as follows: (1) recurrent pterygium; (2) non-RCT study; (3) follow-up time which is shorter than half a year; (4) reviews or clinical case reports.

### Data extraction

For each selected study, two reviewers (WWL and BW) separately extracted and reviewed the relative data. Discrepancies between two reviewers were settled by a third reviewer (YYL).

The data collected were as follows: first author, year of publication, country, type of trials, surgical procedure, postoperative pain, age, number of eyes involved, recurrence and follow-up time.

### Quality of assessment

We assessed the quality of the final selected trials based on the “risk of bias” tool from the Cochrane Handbook 5.1.0 [15]. The following seven aspects about quality of the RCTs were assessed: 1) random sequence generation, 2) allocation concealment, 3) blinding of participants and personnel, 4) blinding of outcome assessment, 5) incomplete outcome data, 6) selective reporting, and 7) other bias. Each item was graded into “low risk of bias”, “high risk of bias” and “unclear risk of bias”. Two reviewers (WWL and BW) separately judged the studies according to this scale and any disagreement was settled by consulting a third reviewer (YYL).

### Outcome measure

All included patients were followed up from the day of operation up to minimum of 6 months postoperatively for recurrence outcome. Any postoperative regrowth of fibrovascular tissue extending from the conjunctiva into the clear cornea was defined to be recurrence. And postoperative pain was also included if it was available.

### Statistical analysis

RevMan software (version 5.3; Cochrane Collaboration, Oxford, UK) was applied to perform statistical analysis. Risk ratios (RRs) were applied when comparing dichotomous variables. We conducted every statistics with 95% confidence intervals (CIs). Chi-square test was used to demonstrate the heterogeneity. When *p* < 0.05 and I2 > 50%, significant heterogeneity was proved [16]. Heterogeneity was considered to be low when I2 ≤ 50%, in which case data were analyzed using the fixed-effects model. Other than that, we used random-effects model [17]. When *P* value is lower than 0.05, we deem it to be statistically significant. Publication bias was measured visually using funnel plots.

## Results

### Result of the search

**Fig. 1:**
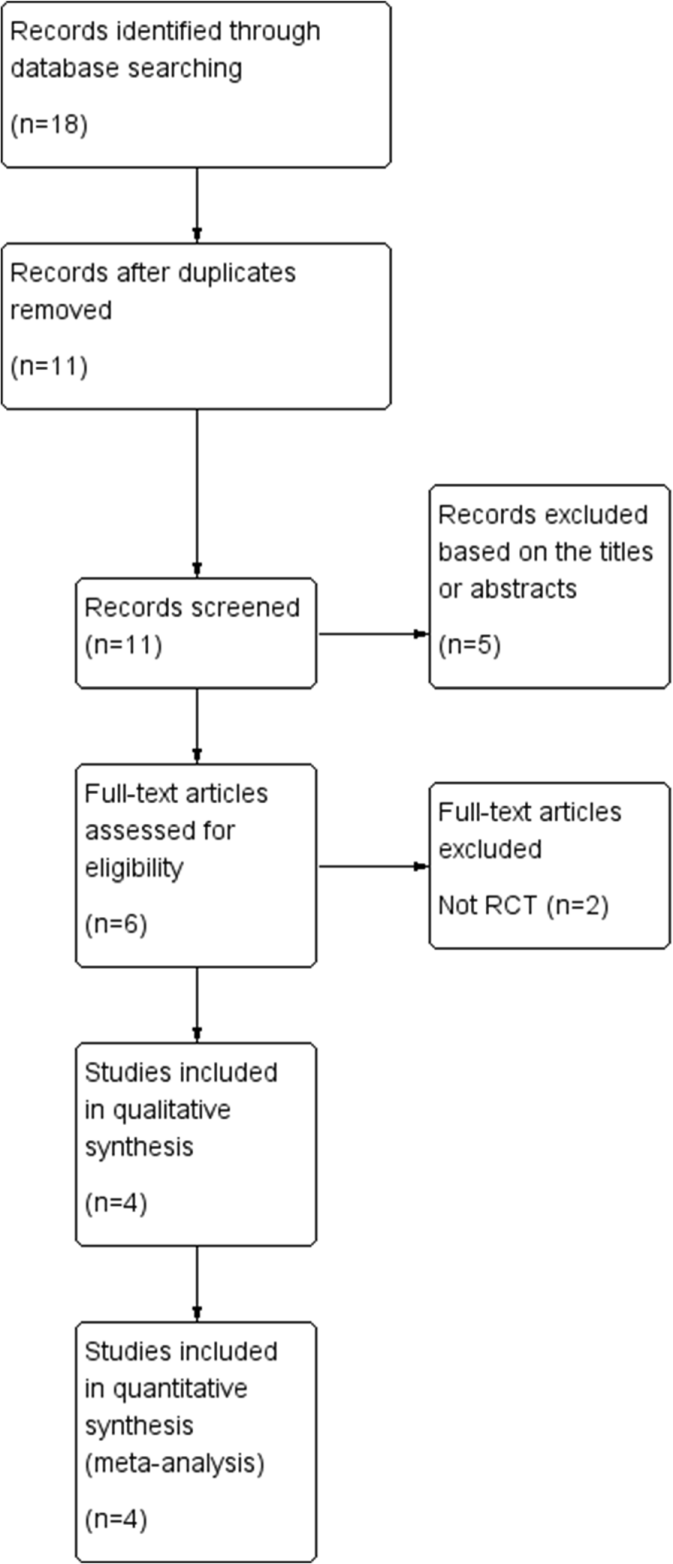
Flow diagram of the study selection process

Altogether, we initially selected 18 studies from online database. Eleven records were reserved when we deleted duplicates. And then 5 records were deleted after scanning titles and abstracts. The remaining 6 records required assessment for eligibility by reading full articles. One record was excluded due to lack of control group. One record was excluded because of its retrospective study type. The other 4 records were involved in the final meta-analysis. We demonstrated the selection process in Fig. 1.

### Study characteristics

In Table 1, we showed the key characteristics of the selected trials. All four involved studies were RCTs. Our meta-analysis enrolled a total of 438 eyes, including 234 in the inferior conjunctival autograft group and 204 in the superior conjunctival autograft group. Of the 4 included studies, the methods to secure the conjunctival graft were 8–0 vicryl suture [13], electrocautery pen [11], 10–0 suture [14] and fibrin glue [12] respectively. Their mean age varied from 47.5 to 58.4 years. The follow-up duration was at least 6 months after operation.

**Table 1:** The characteristics of the selected clinical trials

Author	Year	Country	Study type	Surgery procedure	Postoperative pain	Age		Sample size		Recurrence(%)		Follow-up (month)	
						ICA	SCA	ICA	SCA	ICA	SCA	ICA	SCA
Celeva	2011	Macedonia	RCT	8–0 vicryl suture	NA	48.5 ± 4.4	47.5 ± 7.5	40	40	3 (7.5%)	5 (10.2%)	16.6 ± 4.1	15.5 ± 4.7
Chen	2014	China	RCT	electrocautery pen	less in the ICA group on day 3, 5 (*p* < 0.05).	55.4 ± 10.1	56.2 ± 9.7	40	40	2 (5%)	3 (7.5%)	12	12
Hu	2015	China	RCT	10–0 suture	NA	58. 4 ± 9. 8		129	100	6 (4.7%)	5 (5%)	12	12
Yeung	2013	Canada	RCT	fibrin glue	no statistically different	57.0 ± 15.1	49.5 ± 14.3	25	24	1 (4.0%)	1 (4.2%)	> 6	> 6

### Quality assessment

**Fig. 2:**
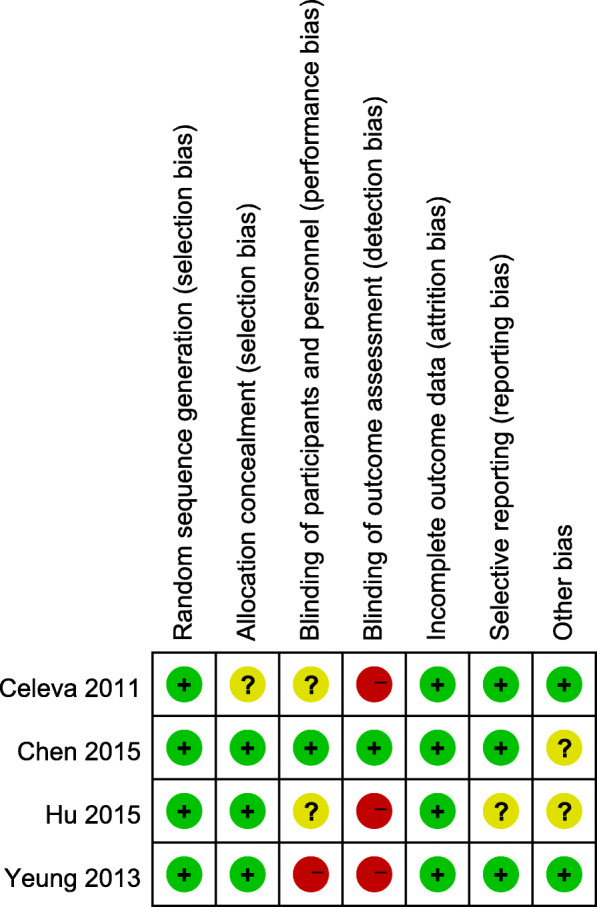
Risk of bias summary

According to the Cochrane risk of bias assessment tool, the risk of bias in included studies was good. The included RCTs had low risk of bias on the whole. A summary of the risk of bias assessment is shown in Fig. 2.

### Outcomes of meta-analysis

#### Pterygium recurrence rate

**Fig. 3:**
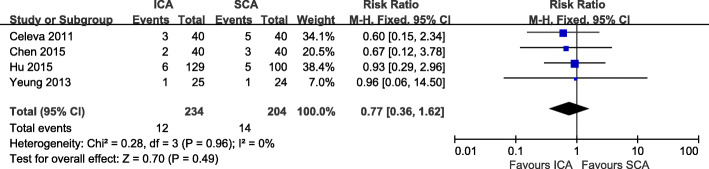
Recurrence Forest plot

Pterygium recurrence rate was compared between the ICA and SCA groups across all the included studies. We found there was no statistical heterogeneity between the two groups (I2 = 0%). A fixed-effects model was applied to analyze the data. With the results of meta-analysis, there was no significantly difference concerning the recurrence rate between the ICA group and the SCA group (RR = 0.77, 95% CI: 0.36 to 1.62, *P* = 0.49; Fig. 3).

#### Postoperative pain

Only Chen [11] and Yeung [12] applied the postoperative pain scale. Since they used different scale systems and the adequate points in the study of Chen were not available, we did not include this outcome in this meta-analysis. However, pain scores were significantly less in the ICA group than the SCA group on follow-up days 3 and 5 according to Chen (*P* < 0.01, *P* = 0.04, respectively). On the other side, pain scores were lower in the study of Yeung on postoperative days 3 (2.6 ± 2.1 for the ICA group versus 3.2 ± 2.5 for the SCA group) and 7 (0.9 ± 1.4 for the ICA group versus 1.1 ± 1.6 for the SCA group), in spite that there were no significant differences (*P* = 0.37 and *P* = 0.69, respectively).

#### Publication bias

**Fig. 4:**
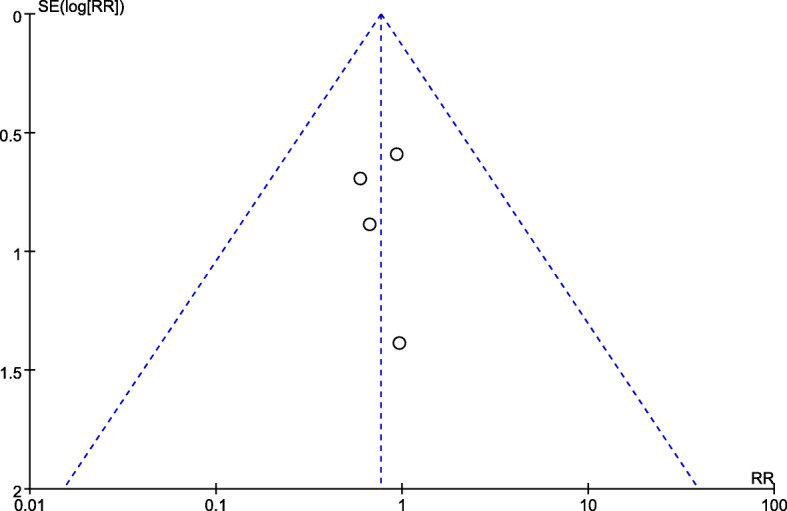
Funnel plot

We analyzed publication bias by using funnel plot. The funnel plots indicated that most points were located in the range of inverted funnel. This proved that no obvious publication bias existed. Therefore, our conclusion was authentic (Fig. 4).

## Discussion

First described in 1985, conjunctival autograft has been extensively applied in the surgery for pterygium. The free autograft incised from nearby conjunctiva will be imposed on the exposed scleral bed after the pterygium is excised. With lower recurrence rates when compared with other operative methods, it is currently thought to be the most effective surgical technique for pterygium treatment. When it comes to the source of the graft, mostly it is obtained from the superior conjunctiva. However, under certain circumstances where the superior conjunctiva area is not available or considered for future therapeutics, the inferior conjunctiva area is more favorable. There are few studies comparing the outcomes between inferior conjunctival autograft and superior conjunctival autograft. Herein, we conducted this meta-analysis to evaluate and compare the recurrence rate between the two groups to generate conclusive evidence.

Our results of this meta-analysis showed that there was no statistically significant difference in the recurrence rate in both groups. All four included RCTs reported the outcome of recurrence. Since recurrence of pterygium after surgical treatment is usually seen within 6 months in most cases, the follow-up time in included studies all last longer than 6 months. All of them showed that both surgery methods were comparative concerning the postoperative recurrence. The inferior autograft is as good as superior autograft in preventing recurrence. In the study of Chen [11] and Hu [14], the conjunctival autograft contained some limbal cells. Although some researchers hold the opinion that the including limbus in the graft is efficacious [18, 19], there was no conclusive evidence with regard to the superiority of limbal–conjunctival autografts over traditional conjunctival autografts [20–22]. Besides, the extra risk of limbal damage at the original site is worthy of more consideration. The complete closure of the excision site with relatively normal conjunctival tissue provides a ‘fire-break’ to the proliferation and advancement of residual abnormal tissue, both conjunctival and episcleral, towards and across the limbus [23]. This theory explains the low recurrence of conjunctival autograft recurrence and equivalence of postoperative recurrence in both groups.

As for the postoperative pain, only Chen [11] and Yeung [12] applied the postoperative pain scale. Since they used different scale systems and the adequate points in the study of Chen were not available, we did not include this outcome in this meta-analysis. It was believed that pterygium excision with inferior autograft generated less postoperative pain and discomfort, for the reason that the upper eyelid had a greater range of motion than lower eyelid, which might produce more ocular surface inflammation postponing the recovery of corneal epithelial [24]. Taking into all these aspects, it is feasible that patients with ocular surface problems using an inferior conjunctival autograft may have a better physical feeling than using a superior conjunctival autograft.

To our knowledge, this is the first meta-analysis to compare the recurrence rate of inferior conjunctival autograft with that of superior conjunctival autograft. Nevertheless, when we use the results of our meta-analysis in clinic, we should bear in mind that our research also carries some limitations. First and foremost, this meta-analysis was limited to researches issued in indexed journals. Thus, no unpublished researches were included in our study, which might have a publication bias. Second, only 4 RCTs were included, of which the conjunctival autograft contained some limbal cells in 2 RCTs, which might have a slight effect on the outcome. Moreover, the technique to secure the graft were different in all studies. Third, other outcomes, such as operation time, intraoperative and postoperative complications, graft status, were not included in our meta-analysis.

## Conclusions

In conclusion, this meta-analysis indicated that inferior autograft transplantation had a similar effect on postoperative recurrence rate, compared with superior autograft transplantation for pterygium. The inferior autograft might have a better performance on postoperative pain. For those who may need future glaucoma filtration surgery or have ocular surface disease, it is more advisable to utilize inferior conjunctival autograft. Subsequent RCTs which have more patients participated and longer follow-up time are needed to confirm our conclusions in years to come.

## Supplementary Information

**Additional file 1.**

## Data Availability

The data that support the findings of this study are available in the references [10–13].

## Abbreviations

ICA: Inferior conjunctival autograft
SCA: Superior conjunctival autograft
RR: Risk ratio
RCT: Randomized controlled clinical trial
CI: Confidence interval
