# Supplementary material for: Recurrence rate with inferior conjunctival autograft transplantation compared with superior conjunctival autograft transplantation in pterygium surgery: a meta-analysis
Source: BMC Ophthalmol. 2021 Mar 9;21:131. doi: 10.1186/s12886-021-01889-4 (PMC7941942; doi:10.1186/s12886-021-01889-4)
Supplement: Supplementary file 1 — Additional file 1. [file 12886_2021_1889_MOESM1_ESM.docx]

Search strategy

PUBMED:

((((((("Pterygium"[Mesh]) OR pterygiums[Title/Abstract])) AND (("Conjunctiva"[Mesh]) OR ((((((((Conjunctivas[Title/Abstract]) OR Tunica Conjunctiva[Title/Abstract]) OR Palpebral Conjunctiva[Title/Abstract]) OR Conjunctiva, Palpebral[Title/Abstract]) OR Bulbar Conjunctiva[Title/Abstract]) OR Conjunctiva, Bulbar[Title/Abstract]) OR Plica Semilunaris of Conjunctiva[Transliterated Title]) OR Plicae Semilunares of Conjunctiva[Title/Abstract]))) AND (((((((("Autografts"[Mesh]) OR Autograft[Title/Abstract]) OR Autologous Transplants[Title/Abstract]) OR Autologous Transplant[Title/Abstract]) OR Transplant, Autologous[Title/Abstract]) OR Transplants, Autologous[Title/Abstract]) OR Autotransplants[Title/Abstract]) OR Autotransplant[Title/Abstract]))) AND superior[Title/Abstract]) AND inferior[Title/Abstract]

Cochrane

ID Search Hits

#1 MeSH descriptor: [Pterygium] explode all trees

#2 (pterygiums):ti,ab,kw (Word variations have been searched)

#3 #1 OR #2

#4 MeSH descriptor: [Conjunctiva] explode all trees

#5 (Conjunctivas):ti,ab,kw OR (Tunica Conjunctiva):ti,ab,kw OR (Palpebral Conjunctiva):ti,ab,kw OR (Conjunctiva, Palpebral):ti,ab,kw OR (Bulbar Conjunctiva):ti,ab,kw (Word variations have been searched)

#6 (Conjunctiva, Bulbar):ti,ab,kw OR (Plica Semilunaris of Conjunctiva):ti,ab,kw OR (Plicae Semilunares of Conjunctiva):ti,ab,kw (Word variations have been searched)

#7 #4 OR #5 OR #6

#8 MeSH descriptor: [Autografts] explode all trees

#9 (Autograft):ti,ab,kw OR (Autologous Transplants):ti,ab,kw OR (Autologous Transplant):ti,ab,kw OR (Transplant, Autologous):ti,ab,kw OR (Transplants, Autologous):ti,ab,kw (Word variations have been searched)

#10 (Autotransplants):ti,ab,kw OR (Autotransplant):ti,ab,kw (Word variations have been searched)

#11 #8 OR #9 OR #10

#12 #3 AND #7 AND #11

#13 (inferior):ti,ab,kw AND (superior):ti,ab,kw (Word variations have been searched)

#14 #12 AND #13
